# Interfacial Chemical and Electrical Performance Study and Thermal Annealing Refinement for AlTiO/4H-SiC MOS Capacitors

**DOI:** 10.3390/nano15110814

**Published:** 2025-05-28

**Authors:** Yu-Xuan Zeng, Wei Huang, Hong-Ping Ma, Qing-Chun Zhang

**Affiliations:** 1 Institute of Wide Bandgap Semiconductors and Future Lighting, Academy for Engineering & Technology, Fudan University, Shanghai 200433, Chinaeehuangw@fudan.edu.cn (W.H.); qingchun_zhang@fudan.edu.cn (Q.-C.Z.); 2 Shanghai Research Center for Silicon Carbide Power Devices Engineering & Technology, Fudan University, Shanghai 200433, China; 3 Institute of Wide Bandgap Semiconductor Materials and Devices, Research Institute of Fudan University in Ningbo, Ningbo 315327, China

**Keywords:** high-k material, AlTiO, 4H-SiC, gate dielectric, interface state

## Abstract

The gate reliability issues in SiC-based devices with a gate dielectric formed through heat oxidation are important factors limiting their application in power devices. Aluminum oxide (Al_2_O_3_) and titanium dioxide (TiO_2_) were combined using the ALD process to form a composite AlTiO gate dielectric on a 4H-SiC substrate. TDMAT and TMA were the precursors selected and deposited at 200 °C, and the samples were Ar or N_2_ annealed at temperatures ranging from 300 °C to 700 °C. An XPS analysis suggested that the AlTiO film had been deposited with a high overall quality and the involvement of Ti atoms had increased the interfacial bonding with the substrate. The as-deposited MOS structure had band shifts of Δ*E*_C_ = 1.08 eV and Δ*E*_V_ = 2.41 eV. After annealing, the AlTiO bandgap increased by 0.85 eV at most, and better band alignment was attained. Leakage current and breakdown voltage characteristic investigations were conducted after Al electrode deposition. The leakage current density and electrical breakdown field of an MOS capacitor structure with a SiC substrate were ~10^−3^ A/cm^2^ and 6.3 MV/cm, respectively. After the annealing process, both the measures of the JV performance of the MOS capacitor had improved to ~10^−6^ A/cm^2^ and 7.2 MV/cm. The interface charge *N*_eff_ of the AlTiO layer was 4.019 × 10^10^ cm^−2^. The AlTiO/SiC structure fabricated in this work proved the feasibility of adjusting the properties of single-component gate dielectric materials using the ALD method, and using a suitable thermal annealing process has great potential to improve the performance of the compound MOS dielectric layer.

## 1. Introduction

The increasing demand for power semiconductor devices using crystalline silicon carbide (SiC), especially 4H-SiC, is driving the continuous development of related research. 4H-SiC has many advantages for applications in power devices. It has a wide bandgap, high breakdown field strength, and high thermal conductivity [1,2,3,4], giving 4H-SiC great potential for application under severe working conditions and the ability to fulfill high power and frequency requirements. MOSFETs are the main type of power device in which using SiC has great advantages, but several issues have been inhibiting the performance of SiC MOSFETs. A silica film formed through heat oxidation is widely applied to the gate dielectric of SiC MOS devices [5,6], but due to their unsatisfactory dielectric qualities, SiC MOS devices have a conductivity, reliability, and gate current leakage that are obviously below theoretical expectations [7]. As such, methods to improve the quality of the gate dielectric layer have been a research area of interest for years.

Besides attempts to refine the widely used SiO_2_ layer, researchers have focused on materials with high dielectric constants. Such high-κ materials have been proposed to solve Si-based MOS dielectric thickness issues, and those which also have wide bandgaps are ideal alternatives to SiO_2_ for SiC substrates. Since Al_2_O_3_ and TiO_2_ have an excellent bandgap width and dielectric constant, respectively, they are two typical high-κ materials. The bandgap of Al_2_O_3_ is very large, almost close to that of SiO_2_, but its dielectric constant is low among high-κ dielectrics [8,9], which makes it relatively susceptible to the problems associated with gate dielectrics with a high electric field strength, similar to those of SiO_2_ dielectrics, such as interfacial defects, oxide traps, and the interface morphology [10,11]. This also leads to the thinning of the physical thickness of the dielectric layer, which increases the leakage current caused by gate tunneling. On the contrary, TiO_2_ has a very high dielectric constant, but its bandgap does not meet the requirements for a SiC substrate gate dielectric and it cannot be applied alone in SiC MOSFETs.

Studies of the use of Al_2_O_3_ as MOS gate dielectrics usually show similar results to those of silica, as the same interfacial issues appear in the Al_2_O_3_ layers as well. Several studies on Al_2_O_3_/SiC MOS devices have applied a thin silica buffer layer to refine the interface [12,13,14], which in fact brings the issue back to the initial state of the SiO_2_/SiC interface. From the point of view of band alignment and gate electric field problems, both Al_2_O_3_ and TiO_2_ are still far from the ideal 4H-SiC MOS gate dielectrics. However, by combining these two dielectric materials into a new dielectric film, the two’s properties are expected to complement one another and result in a composite gate film that has greater potential for performance enhancement.

In this study, an AlTiO layer was fabricated through the ALD process on an n-type 4H-SiC substrate. Besides the as-deposited samples, other samples underwent thermal annealing under different conditions. X-ray photoelectron spectroscopy (XPS), atomic force microscopy (AFM), and ellipsometry (SE) were applied to verify the film deposition quality. The interfacial chemical state, depth profiling, and band alignment of the samples were analyzed based on the XPS results. Al electrodes were deposited on the sample slices in preparation for MOS capacitor performance tests. The leakage current, breakdown field strength, and interface charge, *N*_eff_, of the different AlTiO layers were measured using JV and CV electrical tests.

## 2. Experimental Preparation

A 4-inch 4H-SiC wafer with an n-type SiC epitaxial layer was prepared as a substrate, with a crystal orientation of (0001) (4° offset). The wafer surface was cleaned using a 1:10 HF:DI water solution and then transferred into an ALD chamber for composite gate dielectric deposition. TMA, TDMAT, and water were selected as the precursors of Al, Ti, and O, respectively. The ALD process was carried out at 200 °C. In the repeated ALD cycle, the Al_2_O_3_ layer was first deposited for 9 cycles, with a deposition rate of approximately 1.14 Å/cycle, and the TiO_2_ layer was deposited above the Al_2_O_3_ layer over 5 cycles, with a deposition rate of approximately 0.47 Å/cycle. The final thickness was estimated to have approached 40 nm. Based on the deposition rates of the precursors, the expected Al:Ti stoichiometry was approximately 4.5:1.

The wafer was sliced into 12 × 12 mm^2^ pieces. Three groups of the sliced samples were annealed under a N_2_ atmosphere, and another three groups were annealed using Ar. The samples were annealed at 300 °C, 500 °C, and 700 °C for 30 min, respectively. After material characterization tests, Al electrodes with an area of 200 × 200 μm^2^ were deposited on AlTiO samples with a thickness of 200 nm through magnetron sputtering and prepared for JV and CV characterization. Frequencies of 10 kHz, 100 kHz, and 1 MHz were used to conduct the CV high- and low-frequency tests.

## 3. Results and Discussion

### 3.1. Surface Topographic Analysis

**Figure 1:**
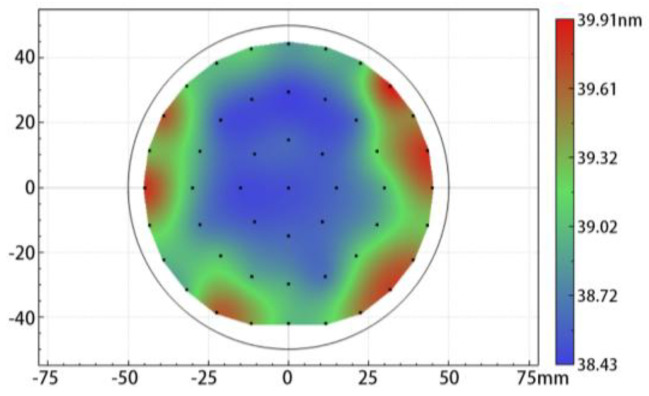
The wafer mapping results based on ellipsometry spectroscopy tests.

An ellipsometry mapping test was carried out on the front side of the deposited AlTiO, as shown in Figure 1, which displays the thickness variation of the as-deposited AlTiO film. The Cauchy model was applied during the raw SE data processing, and the Levenberg–Marquardt algorithm (LMA) was applied to analyze the film thickness. The thickness of the deposited AlTiO layer ranged from 38.4 to 39.9 nm. The thickness distribution near the center of the wafer was more uniform, within the range of 38.4 to 39.0 nm, while the film thickness near the edge was thicker, with a relatively large degree of fluctuation in the film thickness. The AlTiO layer had a refractive index of 1.81 at 532 nm, which was between the refractive indexes of Al_2_O_3_ and TiO_2_.

**Figure 2:**
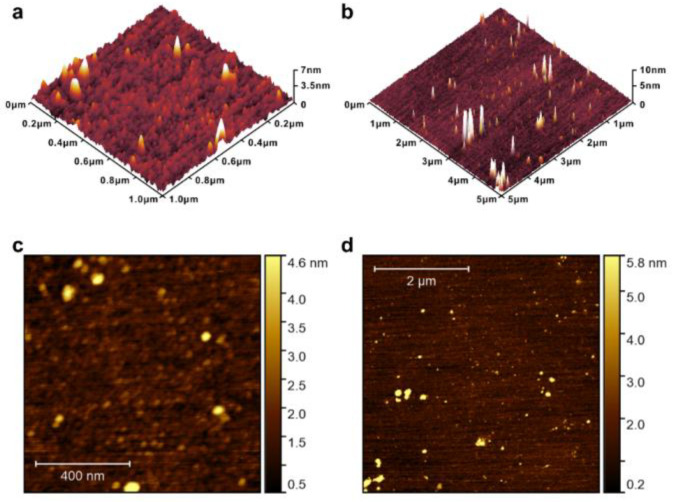
(**a**–**d**) AFM scan of as-deposited AlTiO surface.

The surface topography of the AlTiO films was observed using AFM scans and the roughness was calculated. The AFM scan results are shown in Figure 2, and the vertical coordinates of the three-dimensional topographic map in Figure 2a,b indicate the measured height difference. Besides a number of particles distributed on the surface of the thin AlTiO film samples, the surface was observed to have been uniformly deposited. The roughness of the sample surface, *R*_q_ = 0.77 nm, proved that the thin AlTiO films grown had a high surface flatness. Based on the surface morphology observed using AFM, no obvious crystalline regions were observed on the surface of the films.

### 3.2. XPS Depth Profiling Analysis

**Figure 3:**
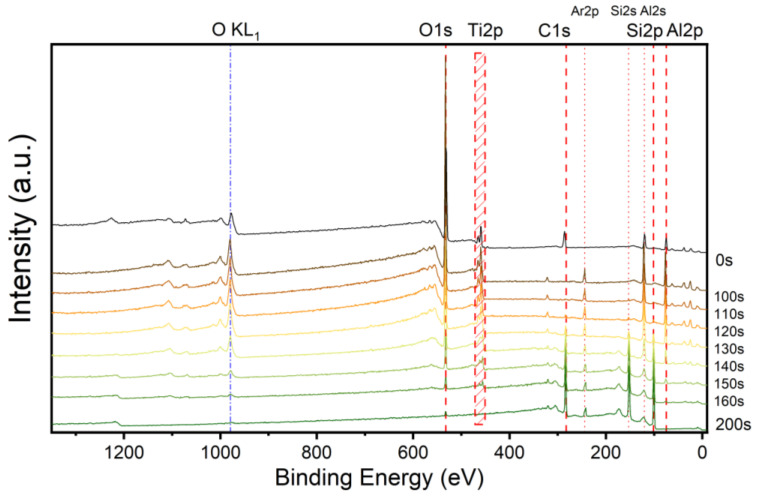
Full XPS survey of as-deposited AlTiO sample at different sputtering depths.

**Figure 4:**
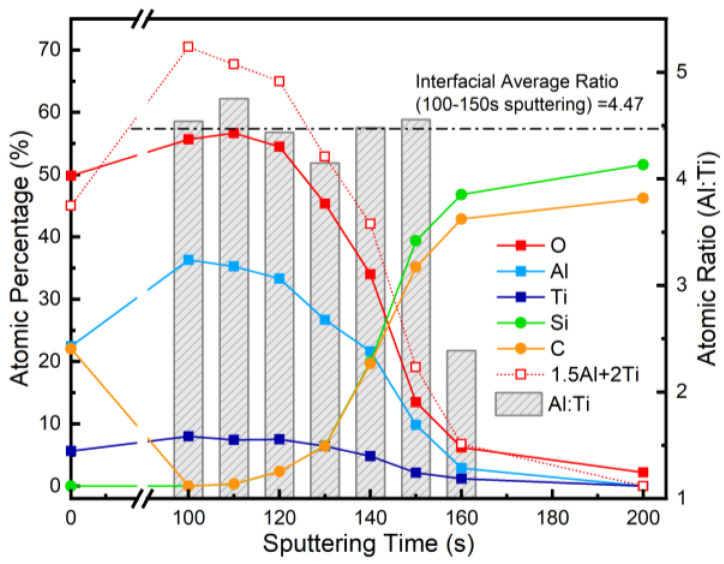
Depth profiling results for as-deposited 4H-SiC/AlTiO heterostructure.

The full-survey XPS spectra in Figure 3 show that the intensity of the Al, Ti, and O peaks dropped dramatically in the etching depth interval of 120–140 s, while the Si and C peaks rose, which indicates that the substrate transitions from the AlTiO film to 4H-SiC were mainly at the etching depth of 120–140 s. Through the peak analysis of the full-survey spectrum, the distribution ratios of various elements at different etching depths were obtained, as shown in Figure 4, in which the atomic ratios of Al and Ti calculated according to the results for the XPS peaks at different depths are given in addition to the ratio of each element at different depths, although the relative error between the low signal intensities of Al 2p and Ti 2p at the etching depth of 160 s was larger than that at a depths of ~140 s. The stoichiometric ratios of Al and Ti were in the range of 4 to 5 with an average of about 4.47, which was consistent with the predicted deposition ratio of the two metal atom precursors during the ALD process.

Based on the comparison of the ratio between Ti, Al, and O, it can be seen that the actual occupancy of the O element was lower than the level predicted by the stoichiometric ratio at the etched positions, which suggests that there was a certain number of Al and Ti atoms with low-valent chemical states within the deposited AlTiO films and that the oxidation reaction involving the oxygen source was incompletely carried out during the ALD growth process. The Al:Ti ratio of the surface (~3.98) was lower than that in the inner sections, presumably due to the order of the ALD process.

**Figure 5:**
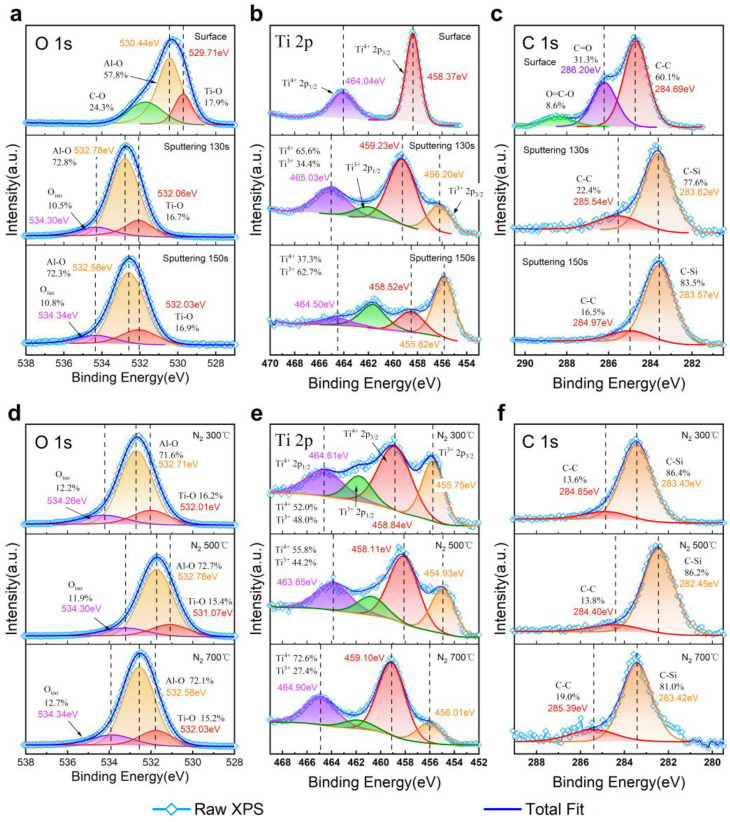
O 1s, Ti 2p, and C 1s XPS spectra of as-deposited sample at different sputtering depths (**a**–**c**) and N_2_-annealed samples at different temperatures (**d**–**f**).

Figure 5a–c show the O 1s, Ti 2p, and C 1s spectra of the as-deposited sample at identical etching depths. In the O 1s spectra, at an etching depth of 130 s to 150 s, the small peaks near 534.3 eV represent isolated O atoms, which suggest interfacial defects and bonding between the substrate and the oxide caused by inner defects, such as oxygen vacancies, Si-O bonding, and -OH groups [15,16]. The Ti 2p spectra show the trend of Ti’s valence varying with the etching depth in near-interfacial regions. The element Ti is present as Ti (IV) in the surface XPS spectra, and the 2p_1/2_ and 2p_3/2_ peaks corresponding to the chemical state of Ti(III) can be observed clearly on both sides of the interface center [17]. The Ti(IV):Ti(III) ratio near the surface (130 s sputtering) was close to 2:1, while there was a lesser proportion of Ti(IV) than Ti(III) near the substrate (at a 150 s etching depth). An analysis of Figure 5b indicates that the influence of SiC on the chemical state of TiO_2_ decreased as the dielectric deposition progressed. The substrate surface may have reacted with TiO_2_ at the beginning of the ALD process and reduced the water source oxidation carried out through TDMAT adsorption to a lower degree.

About 40% of the C 1s spectrum measured at the surface of AlTiO corresponded to an exogenous carbon component, which may have been introduced during the exposure to air before the XPS tests. The C 1s spectra near the interface mainly consisted of C-Si-related peaks corresponding to the SiC component, with a small proportion of C-C or C-Si-O signals. At the interface region depth, near the substrate, there was a lower proportion of these components. These carbon clusters were typically found near the SiC MOS interface, as well as a small amount of substrate oxidation products [18], and the percentage of this component decreased as the etching depth was increased from the 130 s to the 150 s position, so the carbon-related defects should have been enriched in the near-interface region.

**Figure 6:**
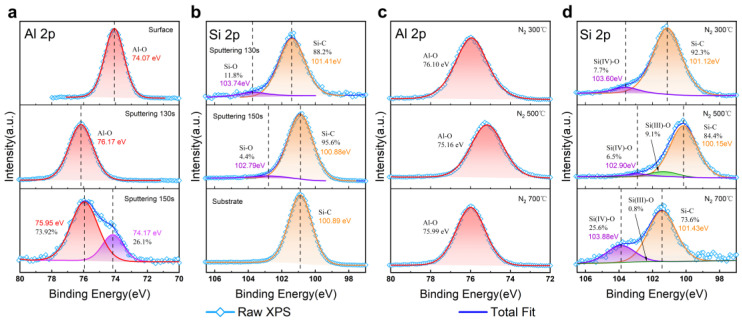
Al 2p and Si 2p XPS spectra of as-deposited (**a**,**b**) and N_2_-annealed (**c**,**d**) AlTiO samples at different sputtering depths.

Figure 5d–f and Figure 6c,d provide the XPS spectra of N_2_-annealed samples at the depth of 130 s etching. The Al-O and Ti-O components in the O 1s spectra had a similar ratio to those in the as-deposited spectra. The proportion of the peak representing the isolated oxygen in O 1s and Ti(III) in Ti 2p slightly increased. This showed a higher level of potential oxygen vacancies compared to the unannealed interface. In the annealed C 1s spectrum annealed with N_2_ at 700 °C, the C-C component made up a higher proportion than in the other samples. This may indicate that high-temperature annealing for 30 min may have induced the formation of carbon clusters in the interfacial region.

Figure 6 shows the spectra of the two elements mainly representing the oxide layer (Al 2p) and the substrate (Si 2p). As shown in Figure 6a,b, which relate to the as-deposited sample, the surface spectrum of Al 2p had a chemical shift of ~2 eV with respect to the inner spectrum of the etched region. This phenomenon may have been related to the involvement of the TiO_2_ component. At etching depths of 140 s and deeper, a small peak near 74 eV emerged, which was assumed to correspond to an isolated Al element [19], suggesting that there was an obstacle to the oxidation process of Al and water in the near-interfacial region, as with Ti. This resulted in the incomplete oxidation of some of the Al atoms, which were left in the near-interfacial region in a low-valence state. The main components in the spectra of Si 2p at all depths were Si-C peaks, and a small peak in the range of 103~104 eV could be observed at the interfacial depth, which corresponded to the Si-O bond. At the depth of the substrate, the proportions of the O element and the Si-O component decreased (with 130 s of sputtering, they decreased to 11.8%), and at the substrate etching depth they became negligible. This indicates that the oxidation level of the substrate was relatively low and that it consisted of oxidate components such as SiO_x_C_y_ in the near-interface region. From 6d, it can be seen that the proportion of Si-O bonding increased as the annealing temperature rose, from 7.7% to over 1/4, and an Si(III) state seemed to appear in the XPS results for annealing at 500 °C and 700 °C, according to our analysis [20]. The intense rearrangement of atoms under high temperatures may have been the main reason for this phenomenon. As such, oxygen formed more bonds with silicon atoms and produced components like SiO_x_C_y_. For N_2_ annealing, a temperature below 500 °C is recommended.

### 3.3. Band Alignment Calculation

**Figure 7:**
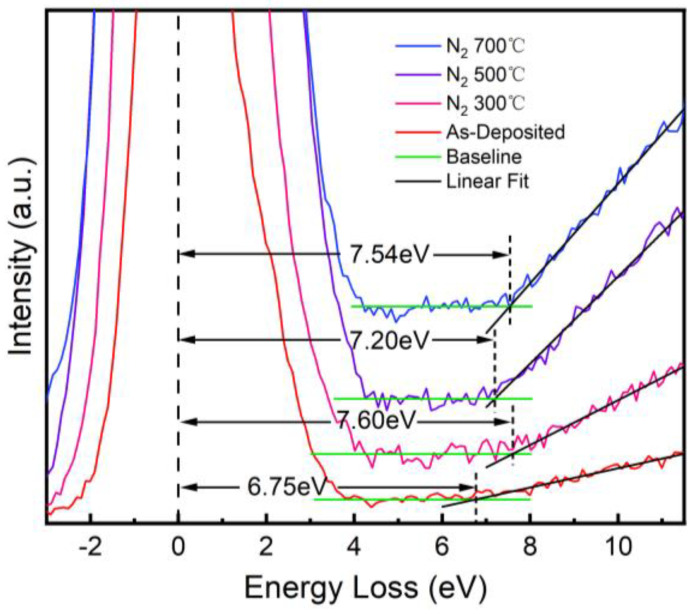
Comparison of bandgap width between as-deposited and N_2_-annealed samples.

The band alignment of the AlTiO/4H-SiC heterojunction was calculated based on the peak fitting results for the XPS spectra. The main peak of Al 2p corresponding to the Al-O bond 2p_3/2_ and the peak of Si 2p corresponding to the Si-C bond 2p_3/2_ were chosen as the reference of core levels for the oxide and the substrate, respectively. The bandgap of the AlTiO layer was determined to be 6.75 eV through the analysis of the EELS spectrum converted from the XPS O 1s results [21], as shown in Figure 7. According to the energy band alignment analysis results, the energy band shifts were ∆*E*_C_ = 2.41 eV and ∆*E*_V_ = 1.08 eV, forming a type I heterojunction. After VBM fitting, we saw that the energy band bending of the sample heterojunction near the interface was low (−0.04 eV), and the sample heterojunction was basically regarded as a flat band in a zero-bias state.

The bandgaps of the AlTiO films of the three annealed samples were calculated using the XPS O 1s spectra for the surface of the samples as well. Compared with that of the unannealed sample, the bandgap of the three samples annealed under a N_2_ atmosphere increased, with the sample annealed at 300 °C showing the highest increase to 7.60 eV. This suggests that annealing significantly increased the gate dielectric bandgap width and there is a possibility to further increase the band shifts to enhance the leakage current suppression ability of the gate.

**Figure 8:**
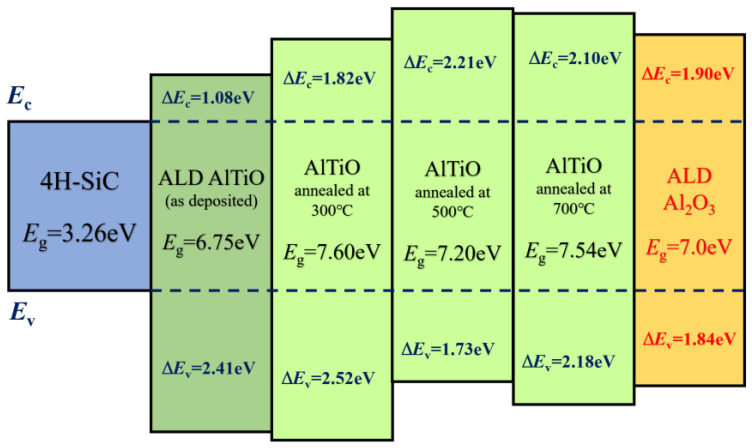
Band alignment between different gate dielectrics and the 4H-SiC substrate. The band alignment of 4H-SiC/Al_2_O_3_ is shown on the right side as a reference [22].

Based on the results from the XPS spectra for different temperatures and an etching depth of 130 s of the annealed samples, the Al 2p_3/2_ (Al-O) and Si 2p_3/2_ (Si-C) peaks were selected to calculate the difference of core levels, and the heterojunction energy level shifts were calculated and are shown in Figure 8. It can be seen that the heterojunctions formed by the three samples were all of type I and had balanced valence band shifts and conduction band shifts. The bandgap width of the annealed AlTiO films increased and the conduction band shift increased to a level of more than 1.8 eV, which was better than that of theAl_2_O_3_ monolithic gate dielectric, indicating that the barrier to the gate tunneling of electrons was improved compared with that of the unannealed sample, and the gate leakage current could be further reduced due to the change of band alignment of the gate heterojunction.

### 3.4. Electronic JV and CV Analysis

**Figure 9:**
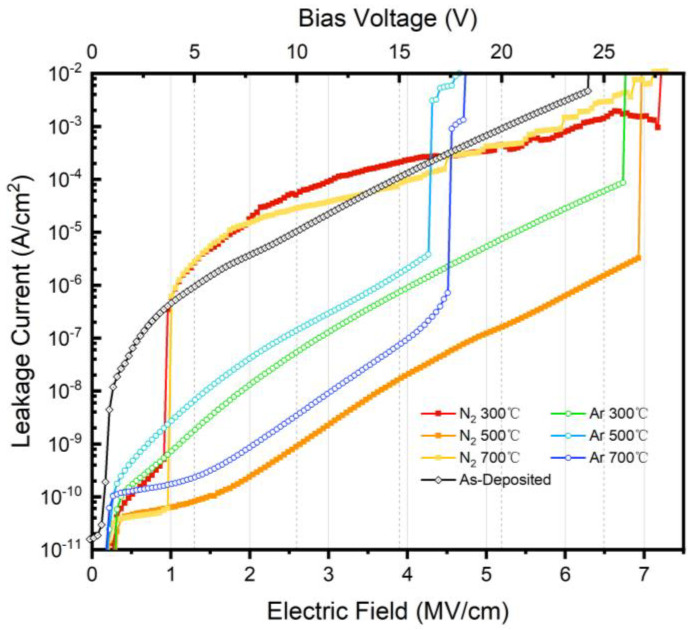
JV results for as-deposited and annealed AlTiO/4H-SiC MOS capacitors.

Keysight B1500 (Keysight Technologies, Santa Rosa, CA, USA) and a Lake Shore probe station (Lake Shore Cryotronics, Westerville, OH, USA) were applied to acquire JV and CV data at room temperature. The JV properties measured after samples were subjected to different annealing conditions are shown in Figure 9. The as-deposited sample had a relatively high leakage current density before breaking down at 6.3 MV/cm [23]. The leakage current density rapidly increased from the order of 10^−8^ A/cm^2^ to 10^−3^ A/cm^2^. Compared with the unannealed samples, annealing the samples at 500 °C under a N_2_ atmosphere or under an Ar atmosphere effectively reduced the gate leakage current to below the breakdown field strength, with the sample annealed at 500 °C under a N_2_ atmosphere reducing the highest leakage current density below the breakdown voltage to the order of 10^−6^ A/cm^2^. From the JV characteristic curve, it can be seen that after the lower-temperature annealing treatment, the breakdown voltage of the samples was also improved. The breakdown electric field strength of the samples annealed at 300 °C under the two annealing atmospheres exceeded that of the as-deposited samples and reached 7.2 MV/cm.

The JV characteristic curves for samples annealed above 500 °C under an Ar atmosphere show that they reached a breakdown voltage below 20 V (or 5 MV/cm), and the leakage current rose significantly with an increase in the positive bias. This indicates that the diffusion of atoms near the interfacial region increased excessively under high annealing temperatures, which induced more interfacial defects and the near-interface oxide traps [24], resulting in the degradation of the quality of the AlTiO gate dielectrics. The annealing treatment using a lower temperature could effectively improve the pressure resistance of AlTiO/4H-SiC and reduce the gate leakage current density.

**Figure 10:**
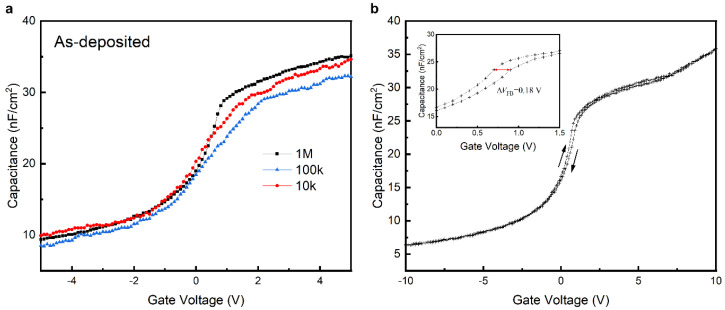
CV scan results for as-deposited AlTiO sample at different frequencies (**a**) and back-and -forth scan curves (**b**).

The CV curves obtained at different frequencies are shown in Figure 10. Among them, the response curve of the sample was the smoothest in the high-frequency band (1 MHz), and there were some fluctuations and poor response stability during the low-frequency test. The capacitance per unit area of the gate in the sample tended to be 35 nF/cm^2^ at the high frequency of 1 MHz under a positive bias. There was no obvious shift in the CV curve center compared with that of the standard gate CV characteristic curve in a zero-bias state. The capacitance density measured at different frequencies was not stable, and there was an overall fluctuation of ±2 nF/cm^2^, which, together with the volatility of the CV curves in the low-frequency state, indicates that the frequency dependence of the sample dielectric was strong, and the interfacial quality was not sufficiently satisfactory according to the following equation:

$$\kappa =\frac{C_{ox}t_{ox}}{\varepsilon_{0}S}$$

where *κ* refers to the relative dielectric constant, *C*_ox_ refers to the gate capacitance, *t*_ox_ refers to the dielectric layer thickness, *S* refers to the area of a single Al electrode, and *ε*_0_ refers to the vacuum dielectric constant. The κ of the sample’s AlTiO film was calculated to be *κ* = 7.8.

The samples underwent CV scanning at a corresponding frequency of 1 MHz with a voltage range of −10~10 V and were scanned forward and backward one time each.

The flat band voltage was determined by the flat band state capacitance:

$$\frac{1}{C_{fb}}=\frac{1}{C_{ox}}+\frac{L_{D}}{\varepsilon_{ox}}$$

where *L*_D_ refers to the Debye length $L_{D}=\sqrt{\frac{\varepsilon_{SiC}kT}{q^{2}N_{D}}}$, determined by the SiC dielectric constant, the Boltzmann constant *k*, the temperature (*T*), the elementary charge (*q*), and the epitaxial layer dopant density (*N*_D_ = 1 × 10^15^ cm^−3^).

In Figure 10b, the direction of the CV scan is identified with arrows, and the flat band voltage shift (Δ*V*_fb_) was measured to be 0.18 V when applying the high-frequency Δ*V*_fb_ result to the following equation [25]:

$$N_{eff}=\frac{C_{ox}{\Delta V}_{fb}}{qS}$$

where *N*_eff_ refers to the interfacial charge density. The *N*_eff_ of the sample was calculated to be *N*_eff_ = 4.019 × 10^10^ cm^−2^.

**Figure 11:**
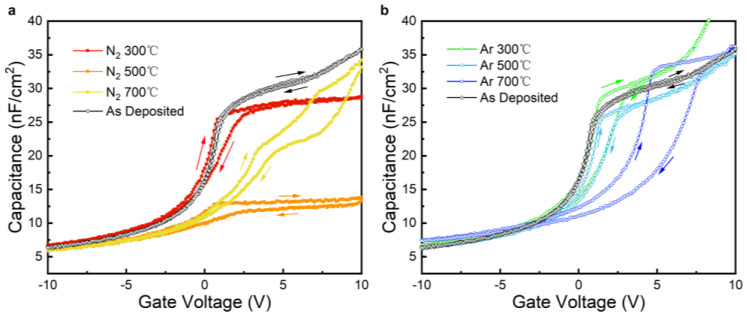
CV scan results for AlTiO samples annealed in N_2_ (**a**) and Ar (**b**) atmospheres under different temperatures. The arrows mark out the sweep direction of the curves.

Figure 11a shows the CV scan characteristic curves of the samples annealed in a N_2_ atmosphere, and it can be observed that the high-frequency capacitances of the annealed samples decreased compared with those of the unannealed samples. The samples annealed at 500 °C had better JV characteristics, but in the CV test, the maximum capacitance in the accumulation region decreased compared with that of the unannealed samples, and it was only 13 nF/cm^2^. The capacitance of an MOS device determines the effective channel mobility, and the higher the gate capacitance, the more carriers it can attract and the lower the channel impedance. The samples annealed with N_2_ showed a general trend that the higher the annealing temperatures, the lower the maximum capacitance in the accumulation region, and the lower the annealing temperature, higher the capacitance of the samples, with the dielectric constant of the samples dropping to 2.9 at 500 °C. The Δ*V*_fb_ of the N_2_-annealed samples increased significantly in comparison to that of the as-deposited sample, and there was a tendency for the Δ*V*_fb_ to increase with a rise in the annealing temperature. Among the three different annealing temperatures, the sample annealed at 300 °C had the smallest Δ*V*_fb_ of 1.30 V, with its *N*_eff_ calculated to be 2.578 × 10^11^ cm^−2^.

Observing the Ar-annealed samples’ CV characteristic curves in Figure 11b, it can be seen that the maximum capacitance in the accumulation region was maintained at a similar level to that of the unannealed sample, except for in the sample annealed at 300 °C, which had a tendency to leak at a gate bias close to 10 V. The Δ*V*_fb_ of the annealed samples was significantly higher than that of the unannealed sample, and the Δ*V*_fb_ of the samples increased significantly with an increase in the annealing temperature, which indicates that the interfacial quality of the samples annealed under Ar conditions exhibited a significant decline with an increase in the annealing temperature and that lower annealing temperatures are required to suppress the degradation of the interfacial quality of the gate dielectrics.

Electrical performance of SiC MOS capacitors using Al_2_O_3_ as the dielectric layer in several similar researches on material have been compared in Table 1. By comparing our results with recent studies on SiC MS capacitors with ALD Al_2_O_3_ dielectrics, even though the performance of the AlTiO dielectric layer produced using the ALD process was not comparatively appealing, the performance regarding the leakage current and defect density of the samples in this work still proved to be above average.

## 4. Conclusions

The ALD-grown AlTiO composite gate dielectrics were uniformly deposited and flat, with a relatively uniform film thickness distribution near the wafer center, and the deposited films were highly compatible with the preparation of amorphous oxide films with a good physical structure. The XPS results suggest the ALD-produced AlTiO compound film had a relatively even component distribution. The chemical states near the interfacial region imply that the AlTiO dielectric reacted more with the SiC substrate than Al_2_O_3_, and the interfacial conditions were much improved compared to those when using TiO_2_. Still, some oxidate components formed during the beginning of the ALD process, including SiO_x_C_y_. The heterojunction formed between the AlTiO film and the substrate was of type I, and the energy band bending near the interface was very small in a zero-bias state, which could effectively inhibit the formation of a gate leakage current due to the energy band arrangement. The annealing process increased the presence of interfacial defects such as oxygen vacancies and carbon clusters as the annealing temperature rose above 500 °C. An increase in the bandgap was achieved for all the annealed AlTiO dielectrics with a range of 0.45–0.85 eV, and the band shift was adjusted to a better position for the MOSFET gate dielectric.

According to the JV test, the gate dielectric of the samples was able to withstand a field strength of 6.2 MV/cm, but the gate leakage current rose significantly with an increase in the forward bias before breakdown, up to the order of 10^−3^ A/cm^2^, so there is a certain amount of room for improvement in terms of the leakage current. Based on the CV test, the response characteristics of the samples in the low-frequency band were not satisfactory, but the overall response characteristics were relatively acceptable for a standard MOS structure. The annealing process, especially Ar annealing, decreased the leakage current density to the order of 10^−6^ A/cm^2^, and annealing at a low temperature of 300 °C or under a N_2_ atmosphere slightly increased the breakdown voltage, making the gate electric field strength tolerance higher. A high annealing temperature also resulted in the excessive rearrangement of interfacial atoms and led to a much higher interfacial state density. To refine the performance of ALD-produced AlTiO dielectrics, Ar annealing at 300 °C or less over a shorter period of heating should be a promising method.

## Figures and Tables

**Table 1:** Electrical performance of SiC MOS capacitors reported in other recent researches.

Dielectric	*E*_BR_ (MV/cm)	*J*_D_ (A/cm^2^)	*D*_it_ (eV/cm^2^)	*N*_eff_ (−*Q*_F_) (cm^−2^)	Ref.	Year
Al_2_O_3_/SiO_2_	-	-	-	2.07 × 10^12^	[26]	2020
Al_2_O_3_	3	4 × 10^−6^	1.5 × 10^11^	4.5 × 10^12^	[27]	2018
Al_2_O_3_	7.5	0.9	1 × 10^12^	2.7 × 10^12^	[28]	2018
Al_2_O_3_	7.5	2 × 10^−4^	5 × 10^10^	1.5 × 10^11^	[29]	2018

## Data Availability

The data will be made available on request.
